# Long-term seasonal forecasting of a major migrant insect pest: the brown planthopper in the Lower Yangtze River Valley

**DOI:** 10.1007/s10340-018-1022-9

**Published:** 2018-07-24

**Authors:** Gao Hu, Ming-Hong Lu, Don R. Reynolds, Hai-Kou Wang, Xiao Chen, Wan-Cai Liu, Feng Zhu, Xiang-Wen Wu, Feng Xia, Miao-Chang Xie, Xia-Nian Cheng, Ka-Sing Lim, Bao-Ping Zhai, Jason W. Chapman

**Affiliations:** 1 College of Plant Protection, Nanjing Agricultural University, Nanjing, China; 2 Centre for Ecology and Conservation, and Environment and Sustainability Institute, University of Exeter, Cornwall Campus, Penryn, TR10 9FE UK; 3 Division of Pest Forecasting, National Agro-Tech Extension and Service Center, Beijing, China; 4 Natural Resources Institute, University of Greenwich, Chatham, Kent, ME4 4TB UK; 5 Rothamsted Research, Harpenden, Herts AL5 2JQ UK; 6 Department of Agriculture, Fisheries and Forestry, Australian Plague Locust Commission, Canberra, ACT Australia; 7 Plant Protection Station of Jiangsu Province, Nanjing, China; 8 Shanghai City Agro-Tech Extension and Service Center, Shanghai, China; 9 Plant Protection Station of Anhui Province, Hefei, China; 10 Plant Protection Station of Guangxi Zhuang Autonomous Region, Nanning, China

**Keywords:** *Nilaparvata lugens*, Windborne insect migration, Atmospheric circulation, Rice pests, Planthopper risk prediction, Western Pacific subtropical high-pressure system

## Abstract

Rice planthoppers and associated virus diseases have become the most important pests threatening food security in China and other Asian countries, incurring costs of hundreds of millions of US dollars annually in rice losses, and in expensive, environmentally harmful, and often futile control efforts. The most economically damaging species, the brown planthopper, *Nilaparvata lugens* (Hemiptera: Delphacidae), cannot overwinter in temperate East Asia, and infestations there are initiated by several waves of windborne spring or summer migrants originating from tropical areas in Indochina. The interaction of these waves of migrants and synoptic weather patterns, driven by the semi-permanent western Pacific subtropical high-pressure (WPSH) system, is of critical importance in forecasting the timing and intensity of immigration events and determining the seriousness of subsequent planthopper build-up in the rice crop. We analysed a 26-year data set from a standardised light trap network in Southern China, showing that planthopper aerial transport and concentration processes are associated with the characteristics (strength and position) of the WPSH in the year concerned. Then, using *N. lugens* abundance in source areas and indices of WPSH intensity or related sea surface temperature anomalies, we developed a model to predict planthopper numbers immigrating into the key rice-growing area of the Lower Yangtze Valley. We also demonstrate that these WPSH-related climatic indices combined with early-season planthopper catches can be used to forecast, several months in advance, the severity of that season’s *N. lugens* infestations (the correlation between model predictions and outcomes was 0.59), thus allowing time for effective control measures to be implemented.

**Electronic supplementary material:**

The online version of this article (10.1007/s10340-018-1022-9) contains supplementary material, which is available to authorized users.

## Key message

- East Asia has experienced a resurgence of serious rice planthopper outbreaks in recent years. In one of the worst-affected areas, the Lower Yangtze Valley of China, the number and timing of brown planthopper immigrants from further south has a major influence on the risk of local outbreaks.
- Here we show how seasonal outbreak risk can be predicted from indices of the intensity of the western Pacific subtropical high-pressure system, a major atmospheric circulation system that drives the synoptic weather patterns affecting planthopper immigration.
- Better prediction allows time for plant protection agencies and other stakeholders to make essential preparations in high-risk seasons.

## Introduction

**Fig. 1:**
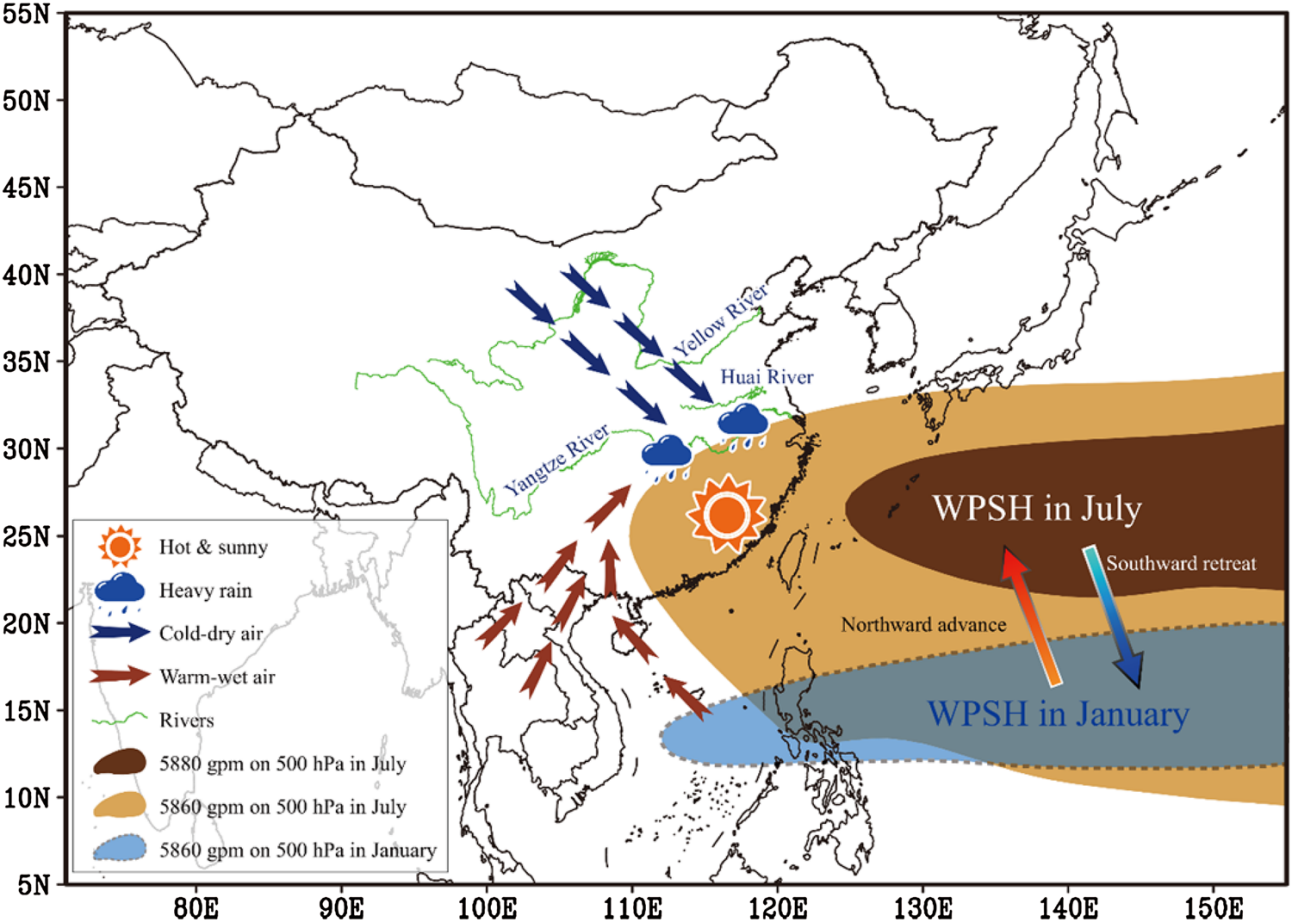
A schematic showing the close relationship between the West Pacific Subtropical High Pressure (WPSH) and the weather in China. The WPSH has regular annual movements, including a northward advance in spring and summer, and a southward retreat in autumn and winter. The subsiding air under the WPSH results in sunny, hot and calm weather, while heavy rainfall occurs just to the north of the WPSH, where the warm-wet airstream and the cold-dry airstream collide. (Color figure online)

The brown planthopper (BPH), *Nilaparvata lugens* (Stål), is the pre-eminent insect pest of rice in Asia, due to devastating feeding damage to the crop (‘hopperburn’) and through the transmission of virus diseases of rice (Bottrell and Schoenly 2012; Cheng 2009, 2015; Otuka 2013; Heong et al. 2015). During heavy outbreaks, BPH can cause almost total crop failure, damaging up to 20 million ha of rice crops annually in China alone (Hu et al. 2011, 2014; Lu et al. 2017). Besides the crop damage itself, there are serious issues associated with insecticide use on planthoppers in China [e.g. high levels of resistance to many insecticidal compounds (Zhang et al. 2014; Wu et al. 2018), disruption of natural enemy control (Cheng 2015) and public health problems (Huang et al. 2015)]. BPH cannot overwinter in temperate regions of China, Korea and Japan; outbreaks in these regions each summer are initiated by a series of five long-range windborne migrations originating in winter-breeding areas in the Indochina Peninsula (Cheng et al. 1979; Kisimoto and Sogawa 1995; Otuka 2013), particularly Central Vietnam (14–19°N; Hu et al. 2017). After the initial invasion of an area, planthopper populations build up in the rice crop and then contribute to the next wave of seasonal northwards expansion. In China, the fourth and fifth waves, in which the breeding area expands from south China into the Lower Yangtze River Valley (LYRV, Fig. 1) (see Box 1 for summary of abbreviations), have the most economic impact as the LYRV is one of the most important rice-producing areas in China. The incidence of outbreaks in the LYRV is more severe and more frequent than in other regions of China (Hu et al. 2011, 2014), but their seriousness varies considerably from year to year. Clearly then, any prediction system relating early-season BPH populations in southern China to the intensity of later outbreaks in the LYRV would be very valuable in providing recommendations to local plant protection workers and to farmers. Ideally, these recommendations could prevent unnecessary insecticide use in seasons of low risk and allow time for plant protection agencies and agricultural advisors to make essential preparations in high-risk seasons.

**Box 1:** List of abbreviations in text (in alphabetical order)

BPH: Brown planthopper (*Nilaparvata lugens*)
CMAP: Climate Prediction Center Merged Analysis of Precipitation
ENSO: El Niño-Southern Oscillation
GLM: Generalised linear model
gpm: Geopotential metres (geopotential height in metres above mean sea level)
LLJ: Low-level jet
LYRV: Lower Yangtze River Valley
NAO: North Atlantic Oscillation
NATESC: National Agro-Tech Extension and Service Centre
NCAR: National Center for Atmospheric Research
NCEP: National Centers for Environmental Prediction
NOAA: National Oceanic and Atmospheric Administration
SSTA: Sea Surface Temperature Anomaly
SSTA(IO–WNP): April–May mean dipolar SSTA difference between the Indian Ocean and the Western North Pacific
WPSH: Western Pacific Subtropical High-pressure

The immigrant density of the planthoppers in a seasonally invaded area (i.e. the LYRV) is dependent on densities in the source areas and on weather conditions which assist or hinder the long-distance nocturnal migrations. Favourably directed, fast-moving winds are the most influential meteorological factor in determining take-off and transport (Chapman et al. 2010, 2015), particularly for small, weak-flying species (Chapman et al. 2011; Hu et al. 2016) such as planthoppers, while rainfall is the most important factor for terminating migrations (Drake and Reynolds 2012). Using a light trap data set from selected plant protection stations over a 26-year period (1978–2003; when a long-established standard ‘black light’ was used in the traps), we explored the effect of two key meteorological factors on the intensity and timing of arrival of the aforementioned fourth and fifth planthopper immigrations. These factors were the development of nocturnal low-level jet (LLJ) south-westerly winds which provide long-distance northward transport for the planthoppers, and zonal rainfall which promotes their aerial concentration and deposition (Watanabe and Seino 1991; Crummay and Atkinson 1997; Feng et al. 2002; Hu et al. 2007; Qi et al. 2010). The position and strength of the western Pacific subtropical high-pressure (WPSH) system, a persistent and large-scale atmospheric circulation pattern (Fig. 1), is known to govern the location of the summer monsoon rains in East Asia (Ding and Chan 2005; Tao and Wei 2006; Ding et al. 2007; Wang et al. 2013). Preliminary studies indicated that outbreaks of BPH in the LYRV are associated with strong WPSH conditions, which promote large immigrations into this region due to the high frequency of suitable LLJs and the location of the rain belt which terminates migration (Lu et al. 2017). In the current study, we further investigated the relationships between latitudinal movements of the WPSH and the spatiotemporal distribution of south-westerly LLJs and rainfall belts which, in turn, will influence BPH migration.

Given the likely impact of the WPSH intensity on planthopper infestations in the LYRV, we then considered indices which may provide earlier indications of WPSH intensity in a given year. The best predictor proved to be the sea surface temperature anomaly (SSTA) index: SSTA(IO–WNP), i.e. the April–May mean dipolar SSTA difference between the Indian Ocean (IO) and the Western North Pacific (WNP). Our models provide a significant improvement to the accuracy of planthopper population forecasting in a region where recent outbreaks have been particularly frequent and severe. The provision of early-season forecasts of the risk of outbreaks of BPH can be used to limit the use of chemical insecticides, thus reducing the risk of resistance developing in the pest while simultaneously helping to conserve natural enemy populations.

## Methods

### Light trap data

Daily planthopper catch data from standardised 20-W ‘black light’ (UV) traps located at the plant protection stations of 222 counties in China were obtained from the National Agro-Tech Extension and Service Centre (NATESC), which has been continuously collecting data since 1977. In this study, data from 8 stations (Fig. S1), which have complete data cover from 1978 to 2003, were used in the correlation analyses. From 2004 onwards, a new light trap design was gradually introduced throughout China (over a period of several years) to replace the ‘traditional’ black light traps. The efficiency of the new traps for catching planthoppers was greatly affected, and during exploratory analyses it proved difficult to take account of changes in the light trap type in the regression models (G. Hu, unpublished analyses). Therefore, we have restricted our analyses to the standardised data set (1978–2003) for the models developed to forecast BPH abundance from WPSH intensity and SSTA indices (see below) and have refrained from including later light trap data in our analyses due to its questionable reliability.

### BPH immigration levels and concentration zones

To represent BPH migration activity, light trap catch data from the 222 stations (Fig. S1) from 1 April to 10 August of each year from 1977 to 2003 were extracted, and the summed catch for each 5-day period (Table S1) for each station was calculated. In order to assign any given station as a ‘concentration and landing zone’ (i.e. a station which received a major immigration of BPH), we calculated the 90th percentile value of catches (termed ‘*BPH90th*’) in each of the 26 5-day periods from 1 April to 10 August. We then produced a time series of 702 of these *BPH90th* values (the 26 5-day periods × 27 years, Table S1) which we termed ‘*TS.BPH*’.

As formulated in the classic paper by Cheng et al. (1979), BPH was expected to expand its range each year from overwintering areas in Indochina by up to five cycles of northwards migration. To examine these migration cycles further, the seasonal variation during the years 1977–2003 was decomposed from our time series (*TS.BPH*) by using linear models. The form of this linear model was:

1 $$\hbox{Log}10 \, \left( {TS.BPH + 1} \right) \, = \, \beta_{1} V_{P1} + \beta_{2} V_{P2} + \beta_{3} V_{P3} + \;\cdots + \beta_{26} V_{P26}$$

where *V*_P1_, *V*_P2_, *V*_P3_, …, *V*_P26_ are the 26 periods of 5-day BPH catches between 1 April and 10 August (Table S1) and *β*_1_, *β*_2_, *β*_3_, …, *β*_26_ are the estimates of the model parameters. Each spell of rapid population growth in this seasonal variation of planthopper numbers was defined as a migration cycle.

**Fig. 2:**
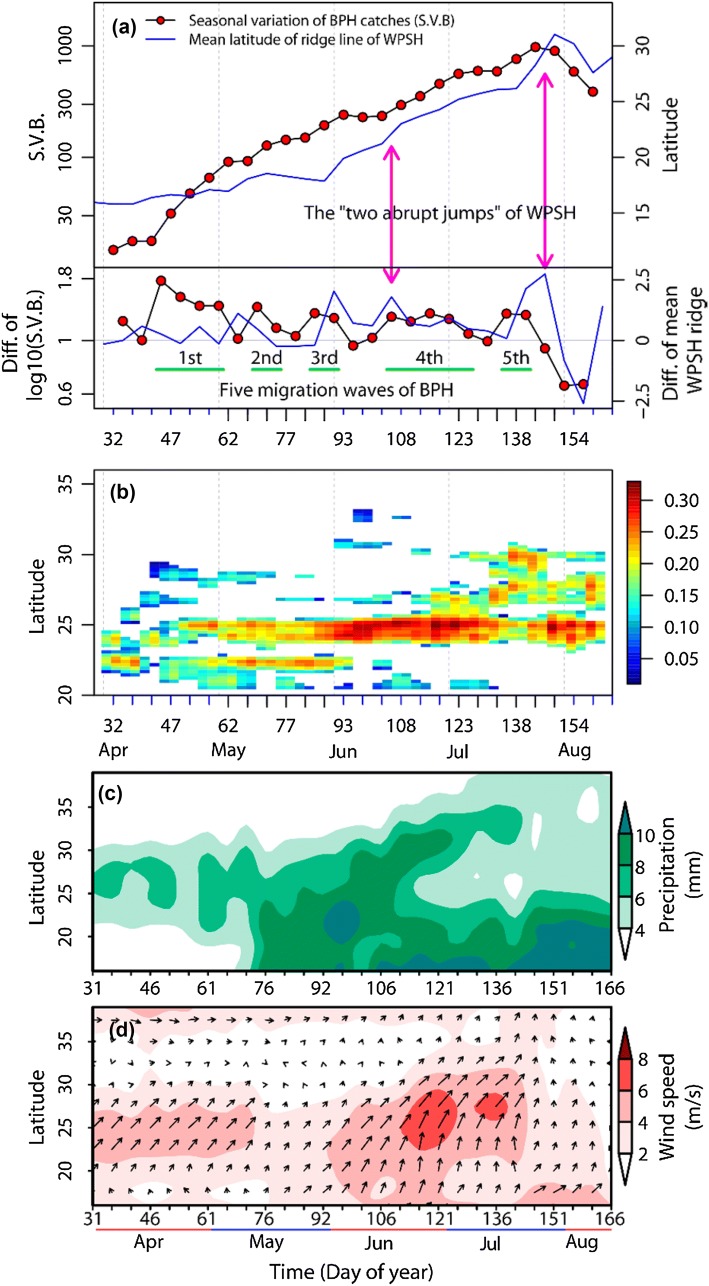
**a** The upper panel shows the mean latitude of the ridge of the WPSH between 110 and 120°E, and the mean seasonal variation in 5-day BPH catches in eastern China (Table S1). The lower panel shows the changes between neighbouring periods; for example for time period *i *+ 1, the value plotted is equal to the value at period *i *+ 1 minus the value at period *i*. The five migration ‘steps’ during the planthoppers’ migrations were interspersed by periods of rapid population growth. The ‘two abrupt jumps’ of the WPSH are meteorologically defined (e.g. Ding and Chan 2005; Tao and Wei 2006; Ding et al. 2007) and denote the beginning and end of the Meiyu season in the Yangtze River Valley. **b** Latitude date cross section of the relative 2-D binned kernel density of the trapping stations (relative density of traps per unit of latitude for each 5-day period) in a BPH concentration zone, based on the data from 222 county plant protection stations between 1977 and 2003. Any given BPH trapping station in any 5-day period was defined as a planthopper ‘concentration and landing zone’ if the number of BPH in the 5-day catches was greater than or equal to the *BPH90th* (i.e. the 90th percentile value in that period of that year). **c** Latitude date cross section of 5-day mean precipitation between 110 and 120°E. **d** Latitude date cross section of 5-day mean winds at 850 hPa height, between 110 and 120°E. (Color figure online)

To explore the seasonal variation in the position of BPH concentration and landing zones, the latitude time cross section of the relative 2-D binned kernel density of stations in a concentration zone was estimated. A heat map showing intensity of BPH outbreaks by latitude and seasonal time period (see Fig. 2b) was generated from raw count data for 5-day periods from all 222 recording sites, using a 2-dimensional kernel smoother to produce density estimates on a regular grid from the irregularly spaced raw data. An outbreak was flagged if a count exceeded the 90th percentile value for the seasonal time period and year in question. The densities shown on the heat map are the probability of an outbreak at the specified time and latitude, minus the probability of any relevant data being available, to allow for uneven data collection. Only outbreak densities which are greater than data availability densities are shown, in order to filter out sporadic density peaks.

### Meteorological data and WPSH indices

The Climate Prediction Center Merged Analysis of Precipitation (CMAP) data, including the monthly and 5-day global-gridded precipitation means since 1979, were obtained from National Oceanic and Atmospheric Administration’s (NOAA) Earth System Research Laboratory (http://www.esrl.noaa.gov/). Their monthly and daily global-gridded data, including the geopotential height and u- and v-winds, were derived from National Centers for Environmental Prediction (NCEP)/National Center for Atmospheric Research (NCAR) reanalysis data from 1948 to 2011. The CMAP and NCEP/NCAR data have a spatial resolution of 2.5°.

Monthly global mean sea surface temperature (SST) since 1854 was obtained from the NOAA Extended Reconstructed SST (version 3b), which has a spatial resolution of 2.0°. Monthly mean North Atlantic Oscillation (NAO) index since January 1950 was obtained from NOAA’s Climate Prediction Center. The sea surface temperature anomaly [SSTA(IO–WNP)] index represents the April–May mean dipolar SSTA difference between the Indian Ocean (IO, 10°S–10°N, 50°E–110°E) and the West North Pacific (WNP, 0°–15°N, 120–160°E). The El Niño-Southern Oscillation development index (ENSO_develop_) denotes the May–March SSTA in the central Pacific (15°S–5°N, 170°W–130°W).

The monthly indexes of WPSH (110°E to 180°E) from 1951 to 2010 were obtained from the China Meteorological Data Sharing Service System (http://cdc.cma.gov.cn/). WPSH is described using five indices: (*V*_A7_, *V*_I7_, *V*_R7_, *V*_N7_ and *V*_W7_ to represent the area, intensity, mean ridge, northern edge and westward extension of the WPSH, respectively).

The five-day mean ridge of the WPSH was calculated using daily NCEP/NCAR reanalysis data. The region of 5860 gpm (geopotential metres) at 500 hPa was used to describe the WPSH. The boundary between the east wind and west wind was defined as the dynamic parameter used to describe the location of the WPSH ridge (Song et al. 2001) and was calculated by the following equation:

2 $$u = 0;\quad\frac{\partial u}{\partial y} = 0$$

where *u* is the speed of zonal wind and coordinate (*x, y*) is its location in the 2-D dimension of u-wind grid data. The location of the five-day mean ridge was calculated by the Grid Analysis and Display System (version 2.0.2, http://grads.iges.org/grads/).

### Regression models of BPH immigration levels in the Lower Yangtze River Valley

As most BPH migrated into the LYRV in July (see *results* and Figs. 2b, 4a), light trap catches of BPH during 1978–2003 from five stations in the LYRV, namely Dongzhi, Huizhou, Gaochun, Nantong and Fengxian (Fig. S1), in July were used to assess immigration into this area (i.e. the response variable *V*_Jul_). The five monthly indices of the WPSH circulation system (i.e. *V*_A7_, *V*_I7_, *V*_R7_, *V*_N7_ and *V*_W7_; Table S2) were the potential explanatory variables used to build a regression model for exploring the quantitative relationship between WPSH and BPH migration (see Box 2 for list of variables).

**Box 2:** List of variables used in the regression models

*TS.BPH* The 90th percentile value of catches in each of the 26 5-day periods from 1 April to 10 August was calculated and termed as *BPH90th*. A time series of 702 of these *BPH90th* values (the 26 5-day periods × 27 years) in 1977-2003 was termed as *TS.BPH*
*V*_P1_, *V*_P2_, *V*_P3_, …, *V*_P26_ Twenty-six periods of 5-day BPH catches between 1 April and 10 August
*V*_Jul_ Light trap catches of BPH during 1978–2003 from five stations in the Lower Yangtze River Valley, namely Dongzhi, Huizhou, Gaochun, Nantong and Fengxian, in July were used to assess immigration into this area
*V*_Maylg_ Light trap data from three stations, i.e. Tianzhu, Quanzhou and Qujiang, were used to represent the abundance of emigrants from the northern South China in May. The sum of light trap catches in these three stations was log-transformed
*V*_A7_, *V*_I7_, *V*_R7_, *V*_N7_ and *V*_W7_ Represent the area, intensity, mean ridge, northern edge and westward extension of the WPSH, respectively. WPSH was principally measured by the location of the 588 geopotential decametre (gpdm) contour lines at the 500-hPa geopotential height field, that is, the region where geopotential height is ≥ 588 gpdm. The area and the mean geopotential height of this region are defined as the area and intensity indices of WPSH. Mean latitudinal position of the WPSH ridge, the longitude of the western-most point and the latitude of the northern-most point were defined as the mean ridge, westward extension and north edge indices of WPSH, respectively
*V*_SSTA(IO–WNP)_ SSTA(IO–WNP), this sea surface temperature anomaly index represents the April–May mean dipolar SSTA difference between the Indian Ocean (IO, 10°S–10°N, 50°E–110°E) and the West North Pacific (WNP, 0°–15°N, 120°E–160°E)
*V*_NAO_ NAO index, based on the surface sea level pressure difference between the Subtropical (Azores) High and the Subpolar Low
*V*_ENSO_ ENSO development index denotes the May-minus-March SSTA in the central Pacific (15°S–5°N, 170°W–130°W)

Previous studies have established that the WPSH variation is primarily controlled by central Pacific cooling/warming and that there is a positive atmosphere–ocean feedback between the WPSH and the Indo-Pacific warm pool ocean. The WPSH intensity index in summer (June–August) can be predicted based on SSTA(IO–WNP), NAO index and ENSO development index (i.e. *V*_SSTA(IO–WNP)_, *V*_NAO_ and *V*_ENSO_ in Table S2) (Wang et al. 2013). These three indices were the potential explanatory variables used to build a forecast model, several months in advance, for planthopper immigration levels in July.

The densities in the source areas have great impact on the immigrant density of the planthoppers in a seasonally invaded area (i.e. the LYRV). Most migrants arriving in the LYRV in July came from northern South China, i.e. northern Guangxi, northern Guangdong, southern Hunan, southern Jiangxi and south-eastern Guizhou (Cheng et al. 1979; Hu et al. 2011). The emigratory adults were the third generation after the initial colonisation of northern South China 2 months earlier, due to their population cycle of macropterous-brachypterous-macropterous forms (Cheng et al. 1979). Light trap data from three stations, i.e. Tianzhu, Quanzhou and Qujiang (see Fig. S1), were used to represent the abundance of emigrants from this source region in May. The log-transformed sum of light trap catches in these three stations formed another potential explanatory variable (*V*_Maylg_) (Table S2) in both regression models.

Data exploration was applied following the protocol described by Zuur et al. (2010). The presence of outliers, auto-correlation in the response variables and collinearity were examined, and the type of relationship was also investigated. Because there was much collinearity in these variables (Fig. S3), each potential parameter was tried in turn and was chosen if it had the best Akaike information criterion (Table S3). Negative binomial generalized linear model (GLM) was applied after the initial Poisson GLMs indicated over-dispersion. Auto-correlation and over-dispersion in the Pearson residuals of the fitted model were checked, justifying the use of a negative binomial GLM.

## Results

As mentioned above, the seasonal expansion of BPH from its overwintering areas in Indochina can be schematised by five cycles of northwards migration interspersed by rapid population growth (Fig. 2a, Table S1). We are concerned here with the factors influencing the last two migration ‘steps’ in China, extending from mid-June to early-July (fourth ‘step’), and from mid-July to late July (fifth ‘step’) (Fig. 2a). In the fourth step, the concentration and landing zones were located in northern South China (north of the Tropic of Cancer in Guangxi and Guangdong Provinces,  ~  25°N) and the LYRV (Figs. 2b, S2d–h). The number of migrants decreased as the migration distance increased and so BPH catches in the LYRV were much smaller than those in northern South China (Fig. S2d–h). The fifth migration step comprises the main movement into the LYRV (Figs. 2b, S2j-k).

### Influence of rainfall and low-level jets on the northward migration

Major zonal rainfall belts with their associated downdrafts, rain and cold temperatures form a barrier to BPH flight and promote concentration and landing (e.g. Crummay and Atkinson 1997; Hu et al. 2007). Just *before* the fourth migration step, the concentration zone was still in South China (Fig. 2b). In mid-June, during the ‘Meiyu season’, the rainfall belt moves to the LYRV (Fig. 2c) causing the concentration zone to shift towards the north in what constitutes the fourth migration step (Figs. 2b, S2d–h). After the Meiyu season is over (around 10 July: Ding and Chan 2005), the rain belt is situated further north again, over the Jiang-Huai Valley (30 to 34°N, i.e. the region between Yangtze and Huai rivers) for 10 days or more (Ding and Chan 2005) (Fig. 2c). The concentration zone consequently shifts to the LYRV—a movement representing the fifth *N. lugens* migration step (Figs. 2b, S2j–k). Thus, the seasonal movement of the East Asian rain belt determines the temporal and spatial distribution of immigrants by alternately allowing and impeding migration (Fig. 2b, c).

**Fig. 3:**
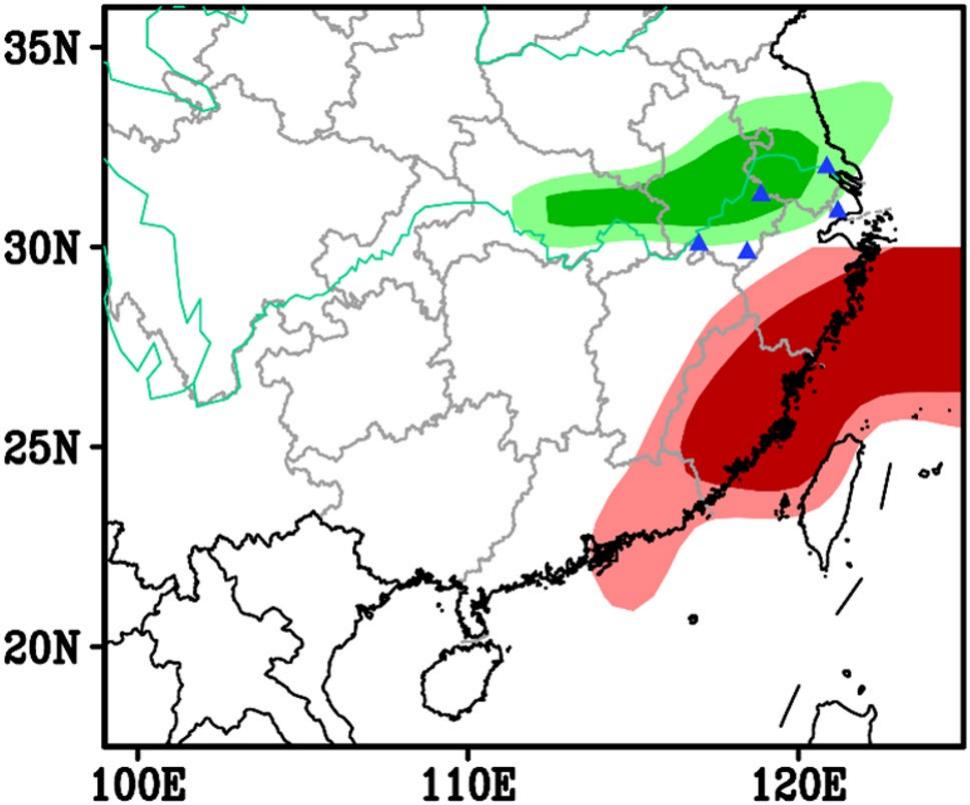
Simultaneous correlation map between BPH immigration levels in the Lower Yangtze River Valley and precipitation (green), and low-level jet (LLJ) days (red) in July. The BPH immigration level is defined as the cumulative sum of light trap catches from five plant protection stations (blue triangles) during 1978–2003. LLJ days are days when the 850 hPa south-westerly wind speed was greater than 12 m/s. The light and dark green/red areas indicate significance at 1 and 5% levels, respectively. (Color figure online)

A spatial map of correlation coefficients between the immigration levels in the LYRV and the July precipitation during 1979–2003 revealed a significant correlation between catches and rainfall in the region immediately to the north (see green-filled region in Fig. 3). The LYRV is located at the southern fringe of the rain belt, which thus forms a natural barrier to migration at this time (Figs. 2c, 3).

The northward migration process itself is facilitated by the development of strong south-westerly winds, particularly the development of nocturnal LLJs which provide rapid aerial transport (Watanabe and Seino 1991; Feng et al. 2002; Qi et al. 2010). Before the fourth migration step, strong south-westerlies were confined to South China but after mid-June the zone of south-westerlies expanded to the north, and the winds strengthened (Fig. 2d) promoting migration to the LYRV. Consistent with this scenario, we found that the number of LLJ days (i.e. those with wind speed ≥ 12 m/s at 850 hPa) in southeast China was significantly correlated with the immigration level in the LYRV in July during 1979–2003 (see red-filled region in Fig. 3).

### Association of the WPSH system with the northward migration

Previous studies have shown that the rain belt distribution in eastern Asia is regulated by the WPSH (e.g. Ding and Chan 2005; Tao and Wei 2006; Ding et al. 2007). Based on results from these studies, we hypothesised that movements of the WPSH would influence the development of south-westerly airstreams and the location of rain belts, and through them BPH movement and concentration (see previous section). The WPSH moves northwards in a stepwise fashion each year, and during summer it exhibits two independent and abrupt movements; the first of these occurs in mid-June when its ridge jumps northward abruptly from South China to the Yangtze River basin, heralding the Meiyu season in the latter region (and much further afield in Korea and southern Japan). The second jump usually occurs in late July, when the WPSH shifts to its most northern position (> 30°N), marking the end of the Meiyu season in the Yangtze River valley and the start of the rains in north China. The association of these movements of the WPSH and the position of the rain belt and the development of the south–westerly airstream are shown in Fig. 2a–d.

The northward shift of the BPH concentration zone coincided with the advance of the WPSH (Fig. 2a). The concentration zone was located north of the WPSH ridge close to the 5860 gpm contour at 500 hPa altitude, whereas the WPSH ridge itself was located south of 30°N (Fig. S2a–l). As the WPSH moved northward in June and July, the BPH concentration zone showed a corresponding northward shift (Fig. S2).

**Fig. 4:**
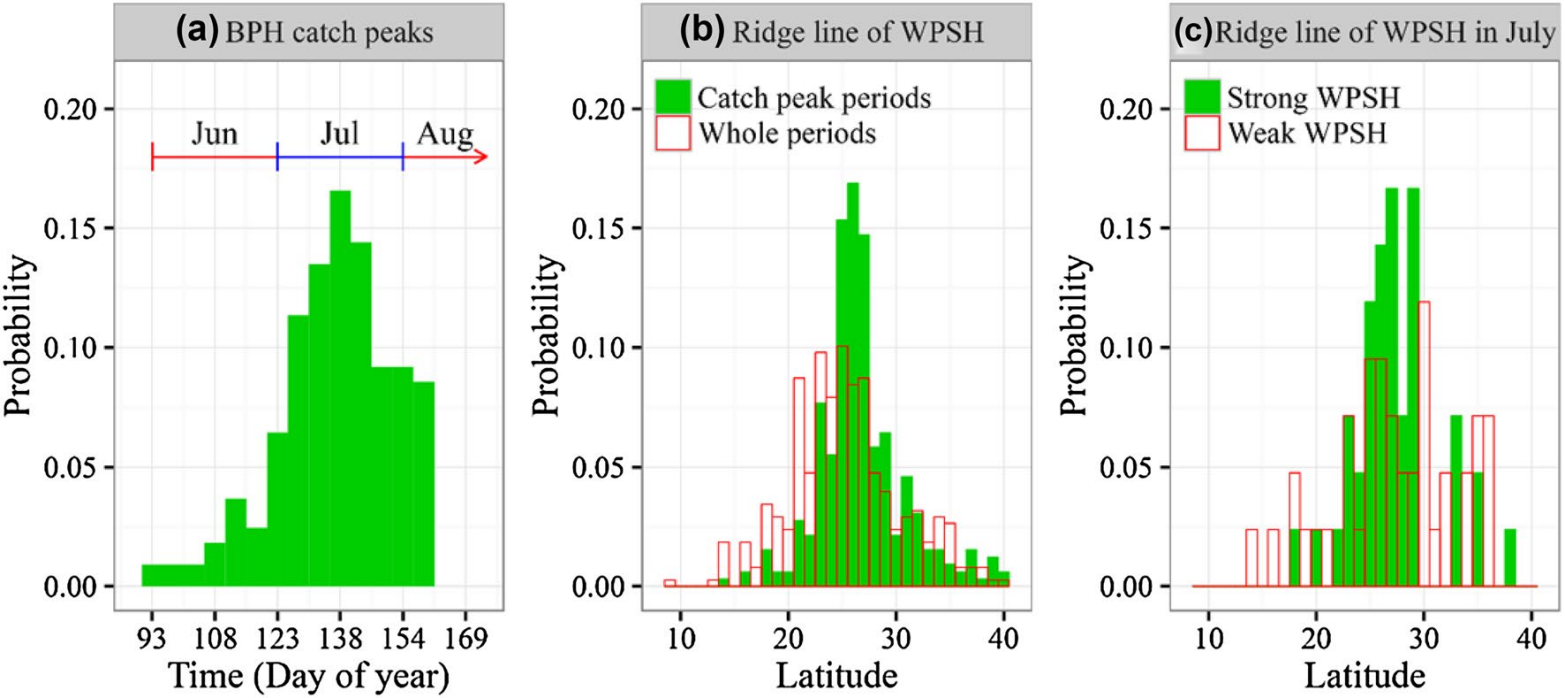
**a** Histogram of 5-day catch peaks of BPH recorded at the 50 stations in the Lower Yangtze Valley before mid-August during 1977–2003. **b** Histogram of the latitude of WPSH ridges when 5-day catch peaks were recorded at these stations (solid green bars), and during the whole period from June to early August (hollow red bars). **c** Histogram of the latitude of WPSH ridges in years with a strong WPSH (solid green bars) and a weak WPSH (hollow red bars). (Color figure online)

We investigated the relationships between the position and intensity of the WPSH and the trap catches arising from the fourth and fifth migration steps. At the time of peak catches of BPH across the 50 stations from the LYRV (Fig. 4a), the average position of the WPSH ridge was 26.53° ± 0.48°N (95% confidence interval, *n* = 326) (Fig. 4b). The distribution of latitudes where ridges were located during these peak catches was considerably tighter (i.e. more concentrated) than that in the period from June to early August as a whole (mean 25.23° ± 0.52°N, (95% CI, *n* = 378)) (compare the green and red bars in Fig. 4b), and these two latitude distributions were significantly different (Fligner-Killeen test: χ^2^ = 18.01, *df* = 1, *p* < 0.001). Therefore, the location of the WPSH and, in particular, its two abrupt jumps (see above) were critical for immigration levels in the LYRV.

To analyse this association further, we distinguished years in which the WPSH intensity in July was classified as ‘strong’ (intensity was ≥ the 3rd quartile value of all WPSH intensities in July 1977–2003), and years when the WPSH intensity in July was classified as ‘weak’ (intensity was ≤ the 1st quartile value) (Table S2). The latitudinal distribution of ridges in strong years (27.20° ± 1.22°N, (95% CI, *n* = 42)) was similar to the spatial distribution of ridges during the period of BPH peak catches in the LYRV (Fligner-Killeen test: χ^2^ = 0.004, *df* = 1, *p* = 0.95) (compare the green bars in Fig. 4b, c). By contrast, the latitudinal distribution of ridges in weak years (27.19° ± 1.79°N, (95% CI, *n* = 42) was significantly different to that during peak catches of BPH (Fligner-Killeen test: χ^2^ = 9.062, *df* = 1, *p* = 0.003) (compare the green bars in Fig. 4b with the red bars in Fig. 4c). Latitudinal distributions of ridges in weak and strong years were also significantly different (Fligner-Killeen test: χ^2^ = 6.434, *df* = 1, *p* = 0.011; Fig. 4c). These analyses indicated that WPSH intensity also has significant influence on immigration.

### Prediction models based on WPSH-related climatic indices

**Fig. 5:**
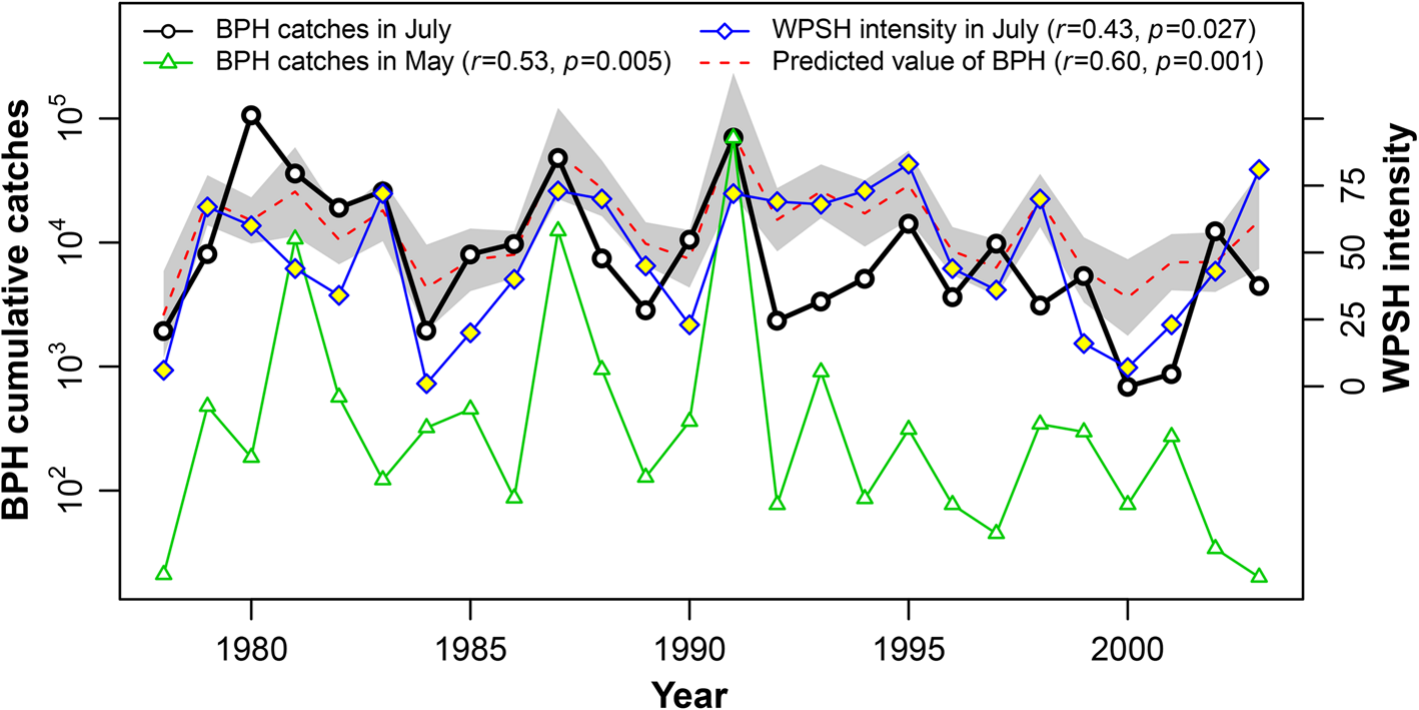
Interannual variations in the immigration levels of BPH in the Lower Yangtze Valley in July (solid black line) in comparison with the catches in South China in May (solid green line) and the WPSH intensity index in July (solid blue line). Also shown is the *predicted* BPH immigration level in July (red dotted line) based on the WPSH intensity in July and catches in South China in May; uncertainty in the predicted values (the forecast standard error) is shown by grey shading. The Pearson’s correlation coefficient (*r*) of July BPH catches and model predictions was 0.60 (*df *= 24, *p *= 0.001). (Color figure online)

Having demonstrated the influence of the WPSH and associated weather factors on immigration into the LYRV, we then developed GLMs to provide forecasts of immigration levels in July—specifically to predict the cumulative light catches in July (*V*_Jul_). The optimal model proved to be:

3 $${\text{Log }}\left( {V_{\text{Jul}} } \right) \, = \, 7.056 \, + \, 0.023V_{I7} + \, 0.224V_{\text{Maylg}}$$

where *V*_I7_ is a WPSH intensity index and *V*_Maylg_ represents light catches in source areas in South China in May. The ‘generalised R^2^’ for this model = 0.34, and the Pearson’s correlation coefficient (*r*) of July BPH catches and model predictions was 0.60 (*df *= 24, *p *= 0.001). The model suggests that an enhanced WPSH indicates an increase in immigration into the LYRV in July and that a model incorporating a measure of WPSH intensity and an estimate of numbers of emigrants in source areas can effectively predict the immigration levels (Fig. 5).

**Fig. 6:**
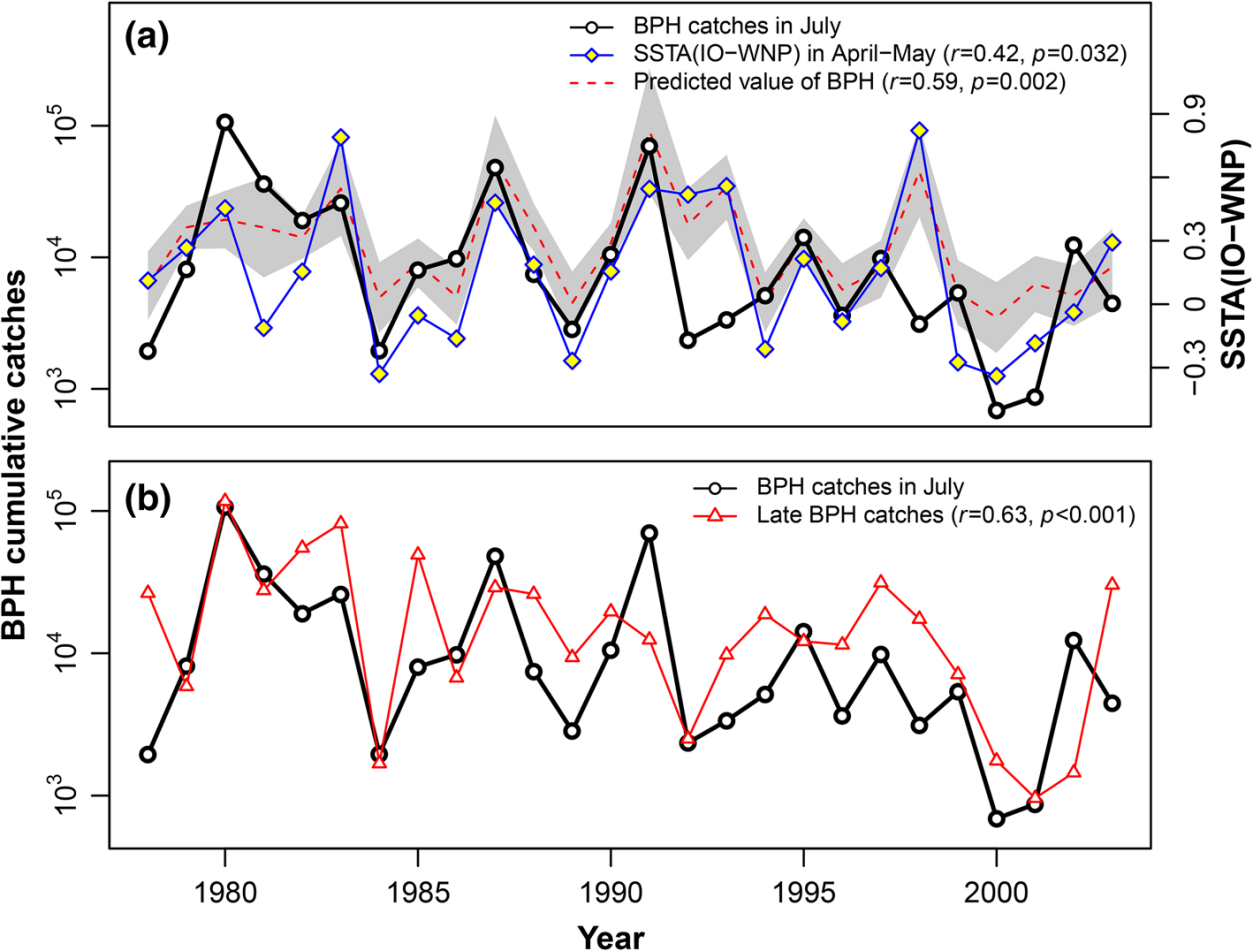
**a** Interannual variations in the immigration levels of BPH in July (solid black line) in comparison with the SSTA(IO–WNP) index for April–May (solid blue line). Also shown is the *predicted* immigration level in July (red dotted line) based on values of the SSTA(IO–WNP) index for April–May and catches in South China in May (see the solid green line in Fig. 5). The Pearson’s correlation coefficient (*r*) of July BPH catches and model predictions was 0.59 (*df *= 24, *p *= 0.002). **b** Interannual variations in the BPH immigration levels in the Lower Yangtze Valley in July (solid black line) were significantly correlated to catches late in the season (solid red line) (*r *= 0.63, *df *= 24, *p *< 0.001). (Color figure online)

We then sought to discover whether a WPSH-related climatic index, such as the ENSO or NAO indices, could provide a longer-range forecast. The most efficient index proved to be the SSTA(IO–WNP) (Fig. 6a). The optimal model was:

4 $${\text{Log}}\left( {V_{Jul} } \right) = \, 7.796 \, + \, 1.904V_{{{\text{SSTA(IO{-}WNP}})}} + 0.232V_{\text{Maylg}}$$

The ‘generalised R^2^’ for this model = 0.41, and the Pearson’s correlation coefficient (*r*) of July BPH catches and model predictions was 0.59 (*df *= 24, *p *= 0.002). There was no advantage in including the other indices in the model as the NAO and ENSO indices had very low correlations with the July catch (Fig. S4). Thus, the magnitude of immigration into the LYRV could be predicted with a reasonable degree of accuracy several months ahead of the arrival date by using the SSTA index and spring catches of BPH from the source area.

Finally, immigration levels in the LYRV in July were significantly correlated with the level of BPH infestation in the late season, i.e. after mid-August (*r* = 0.63, *p* < 0.001) (Fig. 6b). This is interesting because previous studies showed that BPH catches in this region in the late season (late August and early September) are a mixture of locally produced insects and immigrants from further afield (Hu et al. 2014). Nonetheless, the high correlation indicates that high reproduction within this extended area is the main reason for the size of the late infestations and migrations can be considered as internal movement within one population region (see also Hu et al. 2014).

## Discussion

Our previous work has demonstrated that BPH populations in the LYRV originate initially with spring migrations from central Vietnam to South China (Hu et al. 2017), followed by further waves of migration during the summer from South China to the LYRV (Lu et al. 2017). The intensity of the summer migration is related to the strength and position of the WPSH ridge and its associated wind and rainfall patterns (Lu et al. 2017). The results of our current study build upon and validate these preliminary results. Furthermore, they establish that the level of summer immigration into the LYRV is positively correlated with two meteorological factors associated with the strength and position of the WPSH. The first factor is the frequency of suitable nocturnal low-level jets blowing from South China (Fig. 3, red region), which assist windborne transport of BPH from the source region into the study region. The second factor is the intensity of precipitation in the region immediately to the north of the LYRV (Fig. 3, green region), which promotes landing and concentration of migrant BPH just behind the rainfall zone.

We then show that the size of the spring population in South China can be combined with an index of WPSH intensity in July (Fig. 5), to accurately predict the size of the July BPH immigration to the LYRV (Eq. 3 and Fig. 5). This model explicitly demonstrates the link between the WPSH and the immigration level of BPH, but it is of little practical value as it uses meteorological data which is contemporaneous with the arrival of the first wave of migrants, allowing little or no time to make suitable management decisions. What is required is a model which can predict BPH arrival some months ahead of the actual event.

The final stage of our new approach to predicting BPH outbreak intensity during the summer was to determine which (if any) of the teleconnection systems which affect the position and intensity of the WPSH (Wang et al. 2013) could provide *long*-*range* forecasts. We investigated relationships between NAO, ENSO and SSTA(IO–WNP) indices from the spring, and BPH immigration levels in the following summer. Our analyses indicated that the SSTA(IO–WNP) April–May index was the most strongly correlated with the size of the July BPH population (Fig. 6a). When this index is combined with the size of the source population in our final model (Eq. 4), it can be used to reliably forecast the size of the BPH arrival in July (Fig. 6a). As the density of the following generation in August (the time of year when the most severe outbreaks occur) is highly correlated with the abundance of the July immigrants (Fig. 6b), our new model therefore provides a forecast of the risk of a severe BPH outbreak 2–3 months in advance. Our forecasting system can thus provide the information required, within a suitable timeframe, which would enable management decisions to be made and disseminated.

Our model using data from 1978 to 2003 indicates that in order to accurately predict summer immigration of BPH, information on the size of the source population in South China during the spring, and the meteorological conditions during the summer (predicted in advance via the SSTA index) are both required. Anecdotal evidence from the period after our analyses (2004 onwards) provides additional support for the importance of both variables. For example, during this more recent period there have been severe BPH outbreaks in the LYRV in 2005, 2006 and 2007, and in these 3 years there were large source populations in South China and an unusually strong WPSH intensity during July (Lu et al. 2017). However, in 2012 there were also large source populations in South China but the expected serious pest problem in the Yangtze region did not materialise as the summer migrations were impeded by unfavourable winds and heavy rain due to the passage of typhoons (Shi et al. 2014). This was a year with a weak WPSH intensity during July (Lu et al. 2017), and so our model would have correctly predicted no outbreak in 2012. We believe our model could play an important role in the management of rice pests, but in order for this to be possible, it would either be necessary to resume collection of data on BPH population size with the standardised black light trapping network employed in 1977–2003, or develop new models based on the present monitoring system once it has been employed for a period sufficient to standardise the data.

Other climatic indices (ENSO) have been previously used to predict the variations in the occurrence of BPH (Xian et al. 2007). However, the SSTA index we used is directly related to the WPSH and thus has a more obvious and direct connection with the weather influencing BPH migration and population increase than ENSO, which originates from the tropical Pacific Ocean. We believe that our models are therefore less likely to suffer from non-causal associations than some of the earlier work on teleconnection effects (e.g. Xian et al. 2007), because we have shown explicit connections between large-scale atmospheric/sea surface temperature features, seasonal weather and seasonal incursions of BPH into the LYRV.

Early warning of the risk of BPH outbreaks could be extremely beneficial for the management of pests in rice crops. The forecast risk of an outbreak occurring could be disseminated by NATESC via the provincial plant protection stations of each of the 13 rice-growing provinces of southern China, and then onto the county-level plant protection stations and ultimately the growers (e.g. Tang et al. 1994). When the risk is low, this could prevent unnecessary prophylactic spraying of insecticides. For example, in low-risk years farmers following the short-term forecasts from the county plant protection stations can avoid spraying in mid-July and in late July and just apply insecticide in late August; thus, a reduction in insecticide use of the order of 60% seems achievable. Conversely when the risk is high, chemical control could be targeted at the initial July immigration to prevent serious outbreaks in the following generation. BPH has already developed moderate to high levels of resistance to many of the neonicotinoids used against it due to an overreliance on chemical control methods (Wang et al. 2008; Heong et al. 2015; Zhang et al. 2014; Wu et al. 2018), while the indiscriminate use of insecticides also decimates the populations of the natural enemies of BPH. Thus, a pest management system, which incorporates a forecast of outbreak risk, advises the judicious use of chemical insecticides only when required and conserves natural enemy populations, will facilitate control of the pest while simultaneously reducing the risk of resistance developing. Despite years of research on rice pest control, the threat from BPH outbreaks shows no sign of abating (Heong et al. 2015; Hu et al. 2017; Lu et al. 2017). We therefore recommend that a more flexible approach to pest control, based on forecasting of risk and involving significantly reduced quantities of chemical insecticides, is the way forward.

## Authors contribution

GH, JWC and BPZ conceived the research ideas and designed the methodology; XC, WCL, FZ, XWW, FX, MCX and XNC collected the data; GH, MHL, HKW and KSL analysed the data; GH, BPZ, DRR and JWC wrote the manuscript. All authors approve publication.

## Electronic supplementary material

Below is the link to the electronic supplementary material.

Supplementary material 1 (DOCX 1850 kb)
