# Supplementary material for: Long-term seasonal forecasting of a major migrant insect pest: the brown planthopper in the Lower Yangtze River Valley
Source: J Pest Sci (2004). 2018 Jul 24;92(2):417–28. doi: 10.1007/s10340-018-1022-9 (PMC6428905; doi:10.1007/s10340-018-1022-9)
Supplement: Supplementary file 1 — Supplementary material 1 (DOCX 1850 kb) [file 10340_2018_1022_MOESM1_ESM.docx]

**Supporting Information**

**Long-term seasonal forecasting of a major migrant insect pest: the brown planthopper in the Lower Yangtze River Valley**

**Gao Hu, Ming-Hong Lu, Don R. Reynolds, Hai-Kou Wang, Xiao Chen, Wan-Cai Liu, Feng Zhu, Xiang-Wen Wu, Feng Xia, Miao-Chang Xie, Xia-Nian Cheng, Ka-Sing Lim, Bao-Ping Zhai, and Jason W. Chapman**


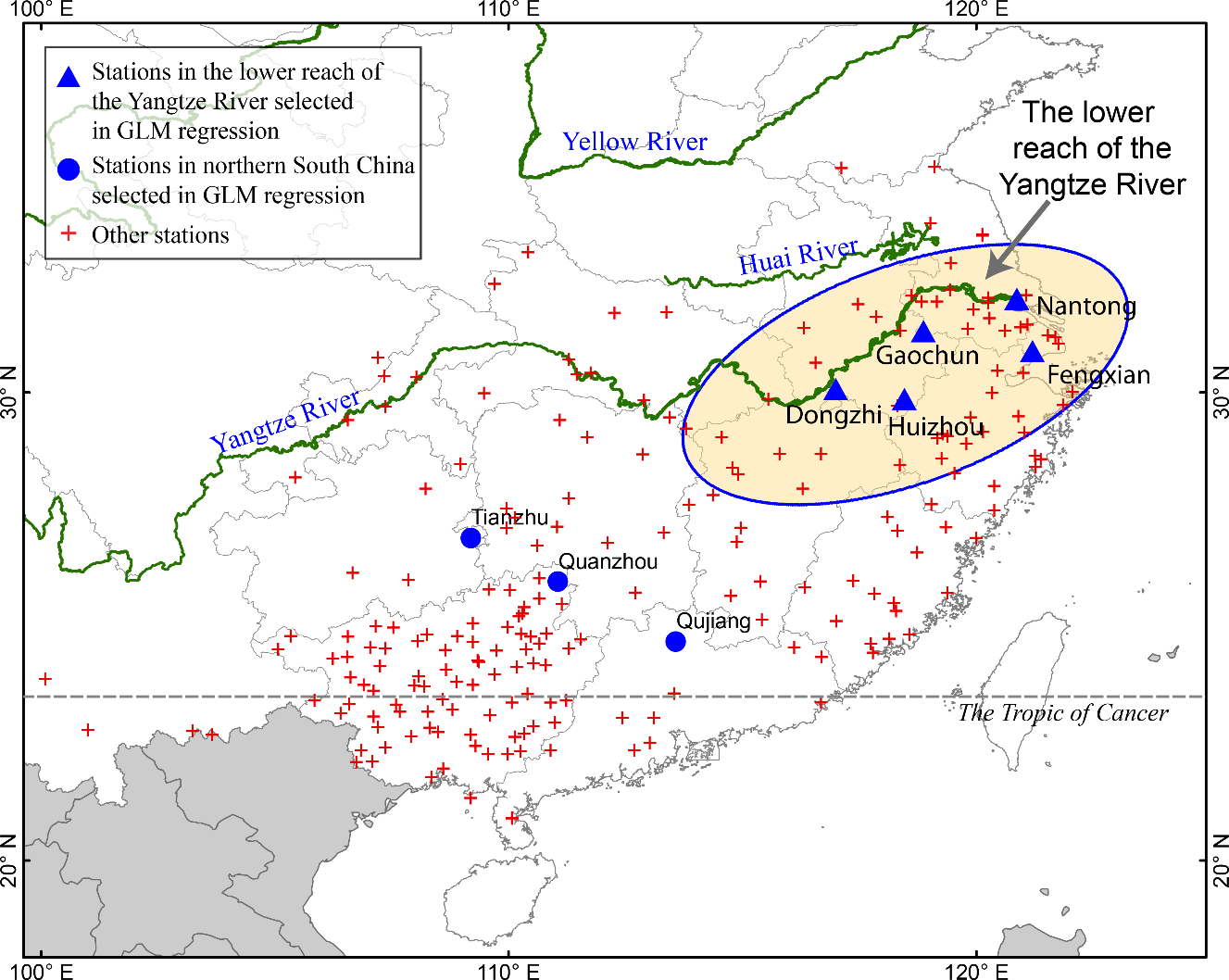


**Figure S1**. Locations of 222 county plant protection stations used to explore the seasonal variation in the position of the planthopper concentration and landing zones. Key stations whose light trap catches were selected to assess emigration from northern South China in May are indicated by three blue circles, while stations used to assess immigration into the Lower Yangtze Region in July are indicated by five blue triangles.


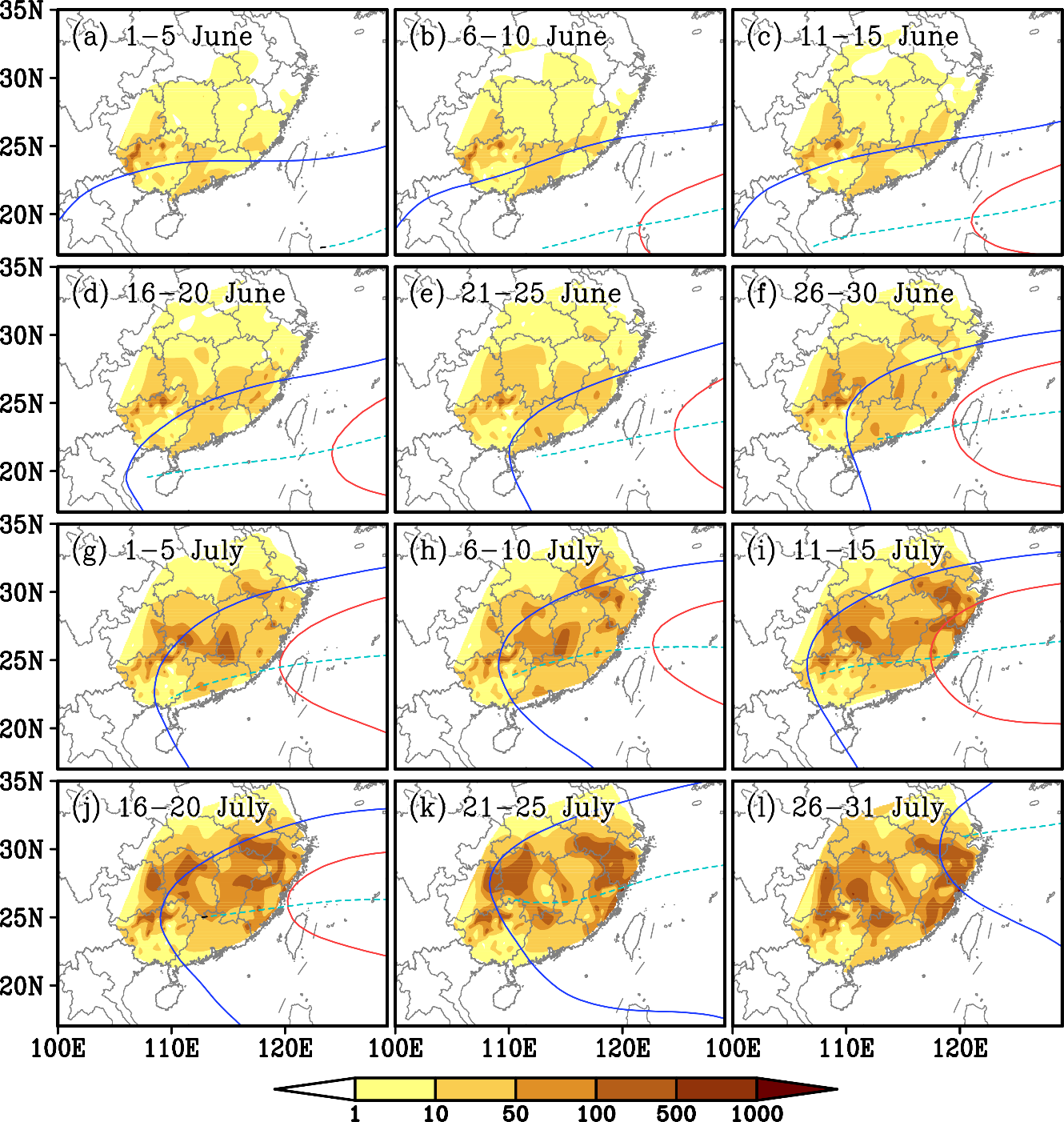


**Figure S2**. Changes in the spatial distribution of *N. lugens* immigration (yellow-brown colored areas) based on mean catches from 1977 to 2003, and the mean West Pacific Subtropical High (WPSH) range, for five day periods in June and July. The *N. lugens* catches from 222 county plant protection stations were interpolated using the ‘natural neighbor’ method in ArcGIS (version 10.2, http://www.esri.com/). The range of the WPSH was presented using a 500 hPa geopotential height contour. The red solid lines denote the contour at 5880 gpm, the blue solid lines denote the contour at 5860 gpm, and the cyan dashed lines denote the WPSH ridges.

**
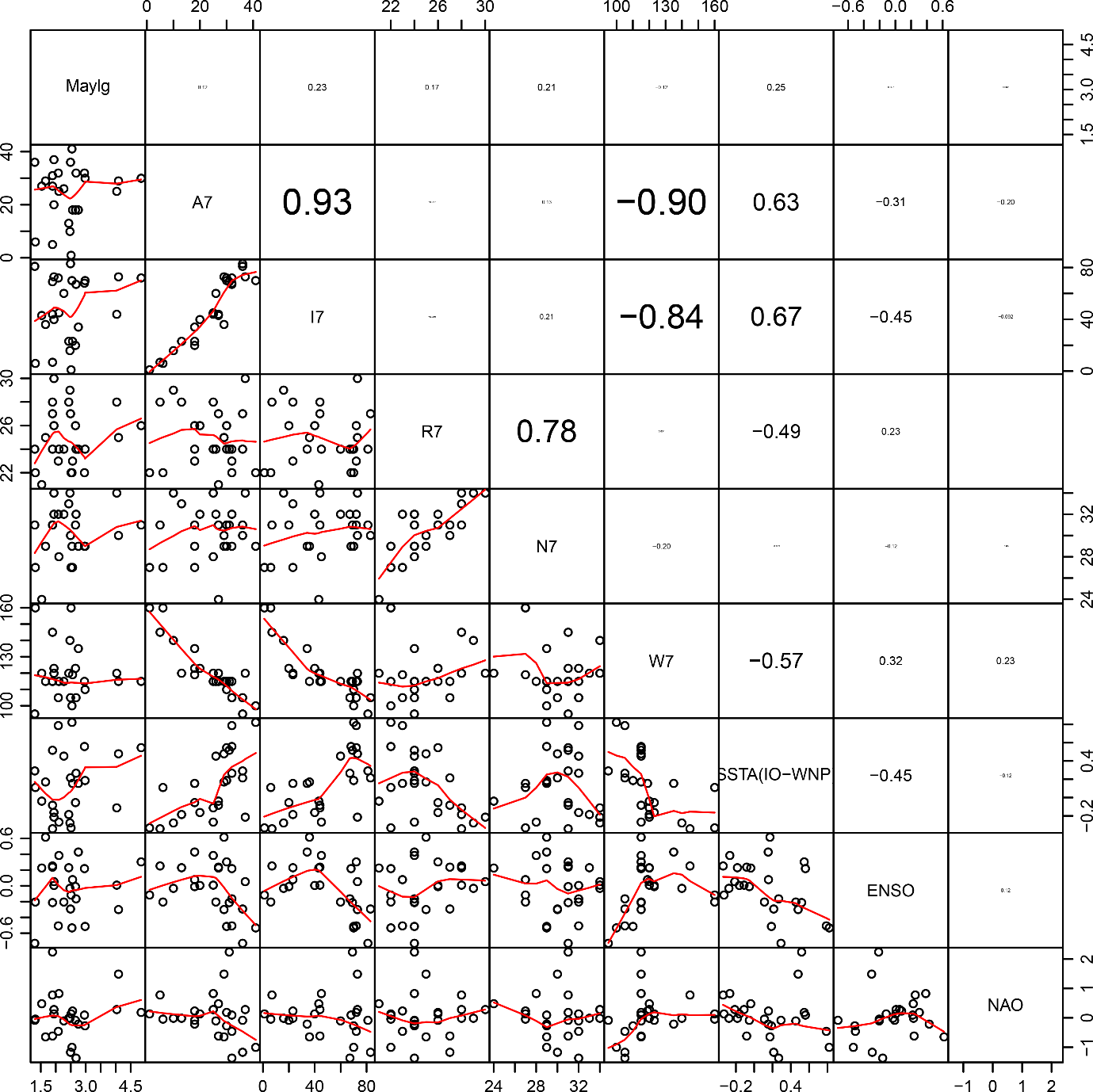
**

**Fig. S3:** Multi-panel scatterplot of all the potential explanatory variables, including the light trap catches in the northern South China (*V*_Maylg_), five monthly indices of the WPSH circulation system (i.e. *V*_A7_, *V*_I7_, *V*_R7_, *V*_N7_ and *V*_W7_) and three WPSH-related climatic indices (i.e. *V*_SSTA(IO-WNP)_, *V*_NAO_ and *V*_ENSO_). The lower / left panels show pairwise scatterplots between each variable, and the upper / right panels contain Pearson correlation coefficients. The font size of the correlation coefficient is proportional to its value.

**
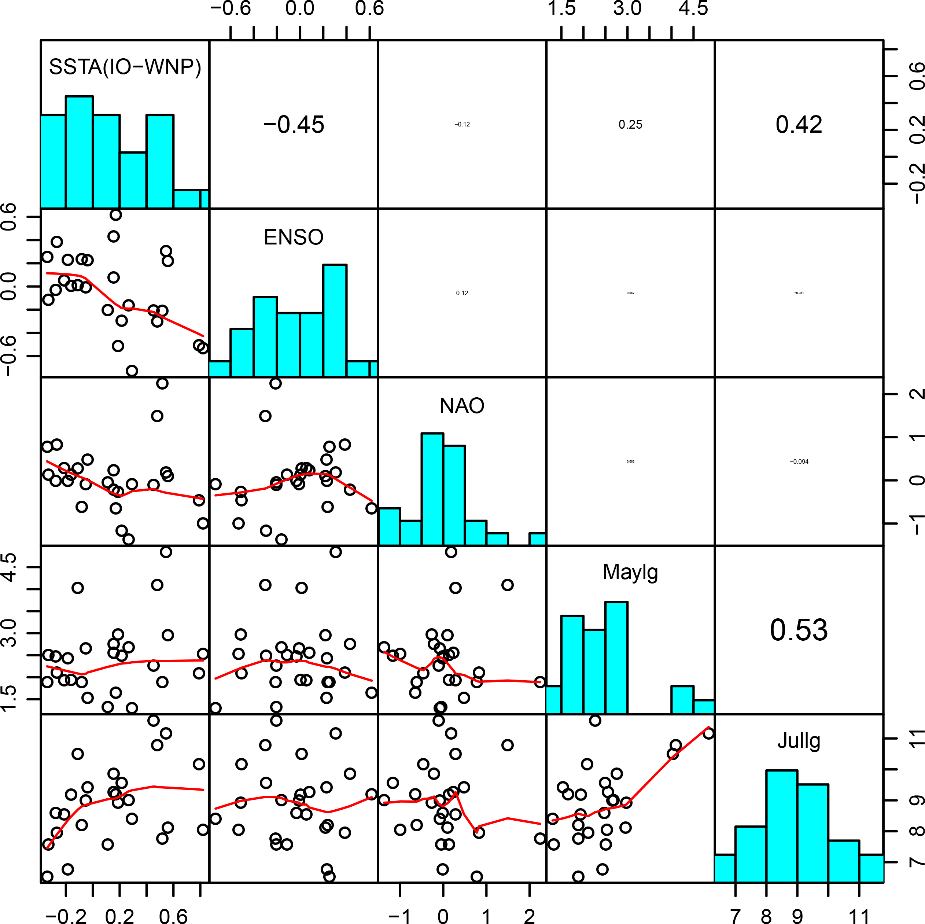
**

**Fig. S4:** Multi-panel scatterplot of the response variable (*V*_Jullg_, i.e. the light trap catches in the Lower Yangtze River) and the potential explanatory variables, including the light trap catches in the northern South China (*V*_Maylg_) and three WPSH-related climatic indices (i.e. *V*_SSTA(IO-WNP)_, *V*_NAO_ and *V*_ENSO_). The lower / left panels show pairwise scatterplots between each variable, and the upper / right panels contain Pearson correlation coefficients. The font size of the correlation coefficient is proportional to its value.

**Table S1:** The seasonal variation in the five-day *N. lugens* catch time series (1977 to 2003) decomposed using linear models. The model was tested by analysis of variance (R^2^=0.971, *F*_26,668_=890, *P*<0.0001).

| Month | Day | Estimate | Std. Error | *t* value | *P* (>\|*t*\|) |
| --- | --- | --- | --- | --- | --- |
| April | 1-5 | 1.1678 | 0.0840 | 13.91 | <0.0001 |
|  | 6-10 | 1.2479 | 0.0805 | 15.49 | <0.0001 |
|  | 11-15 | 1.2492 | 0.0790 | 15.82 | <0.0001 |
|  | 16-20 | 1.4955 | 0.0775 | 19.30 | <0.0001 |
|  | 21-25 | 1.6748 | 0.0790 | 21.21 | <0.0001 |
|  | 26-30 | 1.8177 | 0.0775 | 23.45 | <0.0001 |
| May | 1-5 | 1.9604 | 0.0775 | 25.30 | <0.0001 |
|  | 6-10 | 1.9675 | 0.0775 | 25.39 | <0.0001 |
|  | 11-15 | 2.1061 | 0.0775 | 27.18 | <0.0001 |
|  | 16-20 | 2.1573 | 0.0775 | 27.84 | <0.0001 |
|  | 21-25 | 2.1760 | 0.0775 | 28.08 | <0.0001 |
|  | 26-31 | 2.2881 | 0.0775 | 29.52 | <0.0001 |
| June | 1-5 | 2.3814 | 0.0775 | 30.73 | <0.0001 |
|  | 6-10 | 2.3602 | 0.0775 | 30.46 | <0.0001 |
|  | 11-15 | 2.3716 | 0.0775 | 30.60 | <0.0001 |
|  | 16-20 | 2.4697 | 0.0775 | 31.87 | <0.0001 |
|  | 21-25 | 2.5482 | 0.0775 | 32.88 | <0.0001 |
|  | 26-30 | 2.6595 | 0.0775 | 34.32 | <0.0001 |
| July | 1-5 | 2.7490 | 0.0775 | 35.47 | <0.0001 |
|  | 6-10 | 2.7765 | 0.0775 | 35.83 | <0.0001 |
|  | 11-15 | 2.7727 | 0.0775 | 35.78 | <0.0001 |
|  | 16-20 | 2.8829 | 0.0775 | 37.20 | <0.0001 |
|  | 21-25 | 2.9887 | 0.0775 | 38.57 | <0.0001 |
|  | 26-31 | 2.9561 | 0.0775 | 38.15 | <0.0001 |
| August | 1-5 | 2.7699 | 0.0775 | 35.74 | <0.0001 |
|  | 6-10 | 2.5891 | 0.0775 | 33.41 | <0.0001 |

**Table S2:** Light trap catches of *N. lugens* and meteorological data (1978 to 2003) used in regression models

| Year | Light trap catches | | | | Indices of WPSH in July | | | | | WPSH-related climatic indices | | |
| --- | --- | --- | --- | --- | --- | --- | --- | --- | --- | --- | --- | --- |
|  | Lower Yangtze River Valley* | | Northern South China‡ | | Area | Intensity | Ridge | North  edge | Westward  extension | SSTA(IO-WP) | ENSO | NAO |
|  | July | Late season | May | log(May) | July | July | July | July | July | April-May | May-minus-March | April–May |
|  | *V*_Jul_ | - | *-* | *V*_Maylg_ | *V*_A7_ | *V*_I7_ | *V*_R7_ | *V*_N7_ | *V*_W7_ | *V*_SSTA(IO-WNP)_ | *V*_ENSO_ | *V*_NAO_ |
| 1978 | 1948 | 26495 | 21 | 3.0445 | 6 | 6 | 22 | 27 | 160 | 0.1117 | -0.2041 | -0.045 |
| 1979 | 8140 | 5861 | 477 | 6.1675 | 32 | 67 | 24 | 32 | 105 | 0.2670 | -0.1618 | -1.370 |
| 1980 | 106896 | 117270 | 185 | 5.2204 | 26 | 60 | 24 | 32 | 115 | 0.4533 | -0.2056 | -0.105 |
| 1981 | 36227 | 27709 | 10671 | 9.2753 | 25 | 44 | 28 | 34 | 120 | -0.1120 | 0.0111 | 0.280 |
| 1982 | 19085 | 54988 | 566 | 6.3386 | 18 | 34 | 24 | 29 | 135 | 0.1540 | 0.4309 | -0.215 |
| 1983 | 26010 | 81647 | 122 | 4.8040 | 32 | 72 | 23 | 32 | 105 | 0.7892 | -0.5057 | -0.460 |
| 1984 | 1959 | 1680 | 321 | 5.7714 | 1 | 1 | 22 | 27 | 160 | -0.3297 | -0.1142 | 0.130 |
| 1985 | 8025 | 49258 | 453 | 6.1159 | 18 | 20 | 26 | 31 | 123 | -0.0533 | -0.0081 | -0.085 |
| 1986 | 9780 | 6734 | 87 | 4.4659 | 20 | 40 | 26 | 32 | 123 | -0.1625 | 0.0043 | 0.130 |
| 1987 | 48235 | 29040 | 12414 | 9.4266 | 29 | 73 | 25 | 30 | 115 | 0.4799 | -0.3014 | 1.490 |
| 1988 | 7473 | 26087 | 944 | 6.8501 | 30 | 70 | 24 | 29 | 110 | 0.1874 | -0.5135 | -0.270 |
| 1989 | 2850 | 9364 | 128 | 4.8520 | 25 | 45 | 24 | 28 | 115 | -0.2679 | 0.3860 | 0.830 |
| 1990 | 10555 | 19632 | 361 | 5.8889 | 18 | 23 | 23 | 27 | 119 | 0.1550 | 0.0778 | 0.235 |
| 1991 | 70217 | 12451 | 69753 | 11.1527 | 30 | 72 | 26 | 31 | 115 | 0.5454 | 0.3050 | 0.185 |
| 1992 | 2350 | 2509 | 77 | 4.3438 | 31 | 69 | 24 | 31 | 115 | 0.5182 | -0.2107 | 2.245 |
| 1993 | 3363 | 9777 | 905 | 6.8079 | 32 | 68 | 22 | 29 | 115 | 0.5582 | 0.2189 | 0.095 |
| 1994 | 5140 | 18766 | 86 | 4.4543 | 37 | 73 | 30 | 34 | 120 | -0.2124 | 0.0528 | 0.285 |
| 1995 | 14210 | 12138 | 311 | 5.7398 | 36 | 83 | 27 | 30 | 105 | 0.2144 | -0.2941 | -1.170 |
| 1996 | 3644 | 11490 | 77 | 4.3438 | 27 | 44 | 27 | 31 | 115 | -0.0823 | 0.2369 | -0.615 |
| 1997 | 9828 | 31352 | 45 | 3.8067 | 29 | 36 | 25 | 29 | 115 | 0.1708 | 0.6171 | -0.650 |
| 1998 | 3119 | 17415 | 343 | 5.8377 | 41 | 70 | 22 | 29 | 100 | 0.8211 | -0.5312 | -1.000 |
| 1999 | 5382 | 7103 | 297 | 5.6937 | 10 | 16 | 29 | 34 | 140 | -0.2751 | -0.0307 | -0.015 |
| 2000 | 690 | 1759 | 77 | 4.3438 | 5 | 7 | 28 | 31 | 145 | -0.3396 | 0.2540 | 0.775 |
| 2001 | 871 | 960 | 272 | 5.6058 | 13 | 23 | 28 | 33 | 120 | -0.1848 | 0.2311 | -0.010 |
| 2002 | 12394 | 1451 | 34 | 3.5264 | 27 | 43 | 21 | 24 | 120 | -0.0385 | 0.2270 | 0.480 |
| 2003 | 4473 | 30271 | 20 | 2.9957 | 36 | 81 | 24 | 31 | 95 | 0.2920 | -0.7289 | -0.085 |

* The total catches of five stations, i.e. Huizhou, Dongzhi, Gaochun, Fengxian and Nantong.

‡ The total catches of three stations, i.e. Quanzhou, Qujiang and Tianzhu.

**Table S3:** Results of the GLM models based on a negative binomial distribution.

Model I to test the influence of the West Pacific Subtropical High on the immigration levels in the Lower Yangtze area (*V*_Jul_). Model II to predict *V*_Jul_ from the WPSH-related climatic indices. The Wald test was used to test the statistical significance of regression coefficients; *P*(z) are indicated for significant estimates (****P* < 0.001; ***P* < 0.01; **P* < 0.05). The standard errors of each slope are given in brackets.

| **Models** | **Intercept**  **(**β_0_**)** | **First variable**  **(**β_1_**)** | **Second variable**  **(**β_2_**)** | **Third variable**  **(**β_3_**)** | **Akaike Information Criterion**  **(AIC)** |
| --- | --- | --- | --- | --- | --- |
| **Model I** |  |  |  |  |  |
| Log (*V*_Jul_) = β_0_ + β_1_*V*_Maylg_ + β_2_*V*_A7_ | 6.756***  (0.721) | 0.261*  (0.103) | 0.051**  (0.019) |  | 552 |
| Log (*V*_Jul_) = β_0_ + β_1_*V*_Maylg_ + β_2_*V*_I7_ | 7.056***  (0.634) | 0.224*  (0.102) | 0.023**  (0.008) |  | 550 |
| Log (*V*_Jul_) = β_0_ + β_1_*V*_Maylg_ + β_2_*V*_R7_ | 11.202***  (2.096) | 0.324**  (0.106) | -0.142  (0.084) |  | 554 |
| Log (*V*_Jul_) = β_0_ + β_1_*V*_Maylg_ + β_2_*V*_N7_ | 5.652*  (2.525) | 0.247*  (0.108) | 0.081  (0.085) |  | 553 |
| Log (*V*_Jul_) = β_0_ + β_1_*V*_Maylg_ + β_2_*V*_W7_ | 11.779***  (1.649) | 0.265**  (0.102) | -0.032**  (0.012) |  | 552 |
| Log (*V*_Jul_) = β_0_ + β_1_*V*_Maylg_ + β_2_*V*_I7_ + β_3_*V*_N7_ | 6.312**  (2.371) | 0.213*  (0.103) | 0.022**  (0.008) | 0.028  (0.080) | 552 |
| **Model II** |  |  |  |  |  |
| Log (*V*_Jul_) = β_0_ + β_1_*V*_Maylg_ + β_2_*V*_SSTA(IO-WNP)_ | 7.797***  (0.563) | 0.232*  (0.097) | 1.904***  (0.546) |  | 547 |
| Log (*V*_Jul_) = β_0_ + β_1_*V*_Maylg_ + β_2_*V*_ENSO_ | 7.900***  (0.630) | 0.281**  (0.106) | -0.648  (0.613) |  | 553 |
| Log (*V*_Jul_) = β_0_ + β_1_*V*_Maylg_ + β_2_*V*_NAO_ | 7.830***  (0.633) | 0.300**  (0.106) | -0.268  (0.270) |  | 554 |
| Log (*V*_Jul_) = β_0_ + β_1_*V*_Maylg_ + β_2_*V*_SSTA(IO-WNP)_ + β_3_*V*_NAO_ | 7.768***  (0.564) | 0.238*  (0.097) | 1.867***  (0.551) | -0.091  (0.241) | 549 |
